# Dengue virus infection during window period of consecutive outbreaks in Nepal and assessment of clinical parameters

**DOI:** 10.1038/s41598-023-35928-5

**Published:** 2023-06-07

**Authors:** Ramanuj Rauniyar, Sabita Prajapati, Binod Manandhar, Anup Bastola, Bimal Sharma Chalise, Srijan Shrestha, Chetana Khanal, Machchhendra Thapa, Rajindra Napit, Anup Muni Bajracharya, Shova Shrestha, Anurag Adhikari, Krishna Das Manandhar

**Affiliations:** 1 Central Department of Biotechnology (CDBT), Tribhuvan University, Kirtipur, Kathmandu, Nepal; 2 Department of Mathematical Sciences, Clark Atlanta University, Atlanta, USA; 3 Department of Tropical and Infectious Disease, Sukraraj Tropical and Infectious Disease Hospital (STIDH), Teku, Kathmandu, Nepal; 4 Department of Molecular Biology and Virology, Centre for Molecular Dynamics Nepal (CMDN), Thapathali, Kathmandu, Nepal; 5 Microbiology Department, Balkumari College, Narayangarh, Chitwan, Nepal; 6 Microbiology Department, Trichandra Multiple Campus, Kathmandu, Nepal; 7 Department of Infection and Immunology, Kathmandu Research Institute for Biological Sciences (KRIBS), Lalitpur, Nepal

**Keywords:** Dengue virus, RNA sequencing, Viral infection

## Abstract

Nepal is an endemic country for dengue infection with rolling of every 3 year’s clear cyclic outbreaks with exponential growth since 2019 outbreak and the virus gearing towards the non-foci temperate hill regions. However, the information regarding circulating serotype and genotype is not frequent. This research discusses on the clinical features, diagnosis, epidemiology, circulating serotype and genotype among 61 dengue suspected cases from different hospitals of Nepal during the window period 2017–2018 between the two outbreaks of 2016 and 2019. E-gene sequences from PCR positive samples were subjected to phylogenetic analysis under time to most recent common ancestor tree using Markov Chain Monte Carlo (MCMC) and BEAST v2.5.1. Both evolution and genotypes were determined based on the phylogenetic tree. Serotyping by Real-time PCR and Nested PCR showed the co-circulation of all the 3 serotypes of dengue in the year 2017 and only DENV-2 in 2018. Genotype V for DENV-1 and Cosmopolitan Genotype IVa for DENV-2 were detected. The detected Genotype V of DENV-1 in Terai was found close to Indian genotype while Cosmopolitan IVa of DENV-2 found spreading to geographically safe hilly region (now gripped to 9 districts) was close to South-East Asia. The genetic drift of DENV-2 is probably due to climate change and rapid viral evolution which could be a representative model for high altitude shift of the infection. Further, the increased primary infection indicates dengue venturing to new populations. Platelets count together with Aspartate transaminase and Aalanine transaminase could serve as important clinical markers to support clinical diagnosis. The study will support future dengue virology and epidemiology in Nepal.

## Introduction

Dengue infection caused by dengue virus (DENV) is one of the most important arthropod-borne tropical viral infections transmitted by *Aedes spp.* with an estimation of 390 million infections annually^1^. The genome of dengue virus consists of approximately 11 kb positive-sense, single-stranded RNA molecule^2^. Three structural proteins (Capsid, Membrane and Envelope) and seven non-structural proteins (NS1, NS2A, NS2B, NS3, NS4A, NS4B and NS5) are encoded by the genome and among the three structural proteins, envelope (E) protein plays a critical role in virus attachment and fusion to the host cell membrane to become infective^3^. There are four serotypes of dengue defined by the inability of individually elicited antibodies to cross-neutralize (DENV1-4) and these serotypes are further divided into distinct genotypes as clusters of DENV with sequence divergence not greater than 6% within the chosen genome region^4^. There are five genotypes of DENV 1 (Genotype I, II, III, IV & V), six genotypes of DENV2 (Asian I, Asian II, Cosmopolitan, American, Southeast Asian/ American & Sylvatic), four genotypes of DENV3 (Genotype I, II, III & IV) and four genotypes of DENV4 (Genotype I, II, III & IV)^5^. All of the four serotypes of dengue have expanded their territories to more areas including Asia, Africa and up to Central and South America^6^.

In Nepal, dengue was first documented on 2004 in a Japanese traveller^7^ which was confirmed to be Genotype 1 of dengue of DENV-2 by Takasaki et al. 2008^8^. The first indigenous circulation of dengue virus infection occurred during 2006 with the circulation of all four serotypes^9^. Since then, annual sporadic clinical cases of dengue are being reported with major outbreaks in the years 2010, 2013 and 2016 with shift of serotype from Genotype V of DENV-1 in 2010^10^ to Cosmopolitan IVa and Asian II genotypes of DENV-2 in 2013^11^. In 2016, re-emergence of DENV1 unknown genotype was found^12^.

Dengue viruses show same clinical manifestations and similar patterns of systemic dissemination with tropism principally for monocytes, macrophages and dendritic cells^13,14^. The pathophysiologic changes that occur during dengue fever are still not fully understood. Thrombocytopenia has always been one of the criteria defined by WHO guidelines as a potential indicator of clinical severity and a number of studies have documented platelet dysfunction in DENV infection^15^. Although dengue is a non-hepatotropic virus, liver injury due to dengue infection is common. Hepatic involvement can be characterized by manifestations of acute hepatitis, hepatomegaly, pain in the right hypochondrium, jaundice and raised aminotransferase levels^16^. Clinical studies documenting hepatic involvement in dengue infection are rare. Also, the hematological parameters like hematocrit and hemoglobin should be monitored during dengue fever. The laboratory diagnostic methods for the confirmation of dengue virus infection may involve detection of the virus, viral nucleic acid, antigens or antibodies, or a combination of the detections. After the onset of illness, the virus can be detected in circulating blood for 4–5 days, therefore, detection of the virus nucleic acid is done by PCR during the early stages of the disease. At the end of the acute phase of infection, serology is the method of choice for diagnosis^15^.

This research discusses the clinical features, diagnosis, epidemiology, serotype and genotype circulation and challenges of DENV infection to explore dengue disease extensively in Nepal. This study mainly aims in finding the circulating serotype of dengue virus in the year 2017–2018 and genotype of 2017 along with the study of immune response in Nepalese population determining whether the infection is primary or secondary. It also highlights the biochemical tests i.e., aspartate transaminase (AST) and alanine transaminase (ALT) which could be considered as the marker to predict the dengue cases but not considered as a compulsory test in Nepal. In the present study, DENV strains of the year 2017 in Nepal were characterized by sequencing the envelope protein coding region. The results shed light on the genotypes of the currently circulating dengue virus and add country-specific information to the DENV sequence database.

## Results

### Circulating dengue serotypes detected by Real-time RT-PCR and Nested PCR

In the study year 2017 (n = 14), multi serotypes DENV-1, DENV-2 and DENV-3 were circulating in Nepal but there were only one serotype DENV-2 in 2018 (n = 12) was detected by Real-Time PCR serotyping kit provided by Center for Disease Control (CDC), USA among the 61 samples (Table 1). Among those PCR positive samples of the year 2017, 14.3% (n = 2/14) were found to be DENV-1, 78.6% (n = 11/14) DENV-2 and 7.1% (n = 1/14) DENV-3 while all the cases (100%, n = 12) of the year 2018 were DENV-2. However, DENV4 was not found in both the years (Table 1).

**Table 1:** Circulation of different serotypes of dengue as detected by PCR (Real-Time PCR and Nested PCR).

Serotype	No. of cases			Districts	Provinces
	2017 (n = 14) (%)	2018 (n = 12) (%)	Total (n = 26) (%)		
DENV 1	2(14.3)	–	2(7.7)	Rupandehi	Lumbini
DENV 2	11(78.6)	12(100)	23(88.46)	Sarlahi, Chitwan, Jhapa, Makwanpur, Kaski, Dhading, Kapilvastu, Syangja, Ramechhap, Kathmandu, Rauthat	Madhesh, Bagmati, Koshi, Gandaki,Lumbini,
DENV 3	1(7.1)	–	1(3.9)	Bardiya	Karnali
DENV 4	–	–	–	–	–

**Figure 1:**
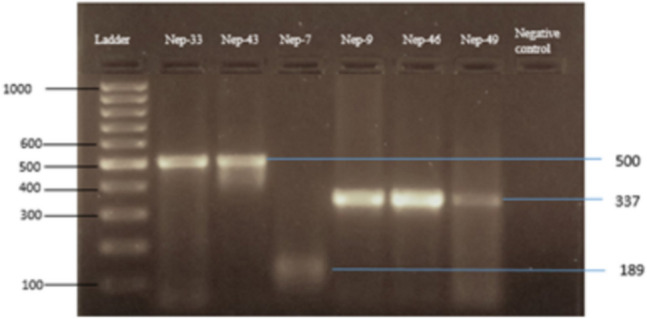
Gel image under UV Transilluminator of the second round nested PCR of envelope gene that gave 500 bp, 337 bp and 189 bp amplicon corresponding to the expected size of DENV-1, DENV-2 and DENV-3 respectively. 100 bp ladder [Thermo Scientific Generuler 100 bp DNA ladder, ready to use (Cat. No. #SM0243)] was run along with the PCR product.

Serotype-specific nested PCR performed in the Real-Time PCR positive samples (n = 26) expressed amplicons of varied sizes; 500 bp for DENV-1, 337 bp for DENV-2 and 189 bp for DENV-3 sample (Fig. 1, Supplementary Figure S1). No cases of concomitant infection with more than one serotype were observed.

### Sequence analysis of E-gene and genetic evolutionary linkage by phylogeny

**Figure 2:**
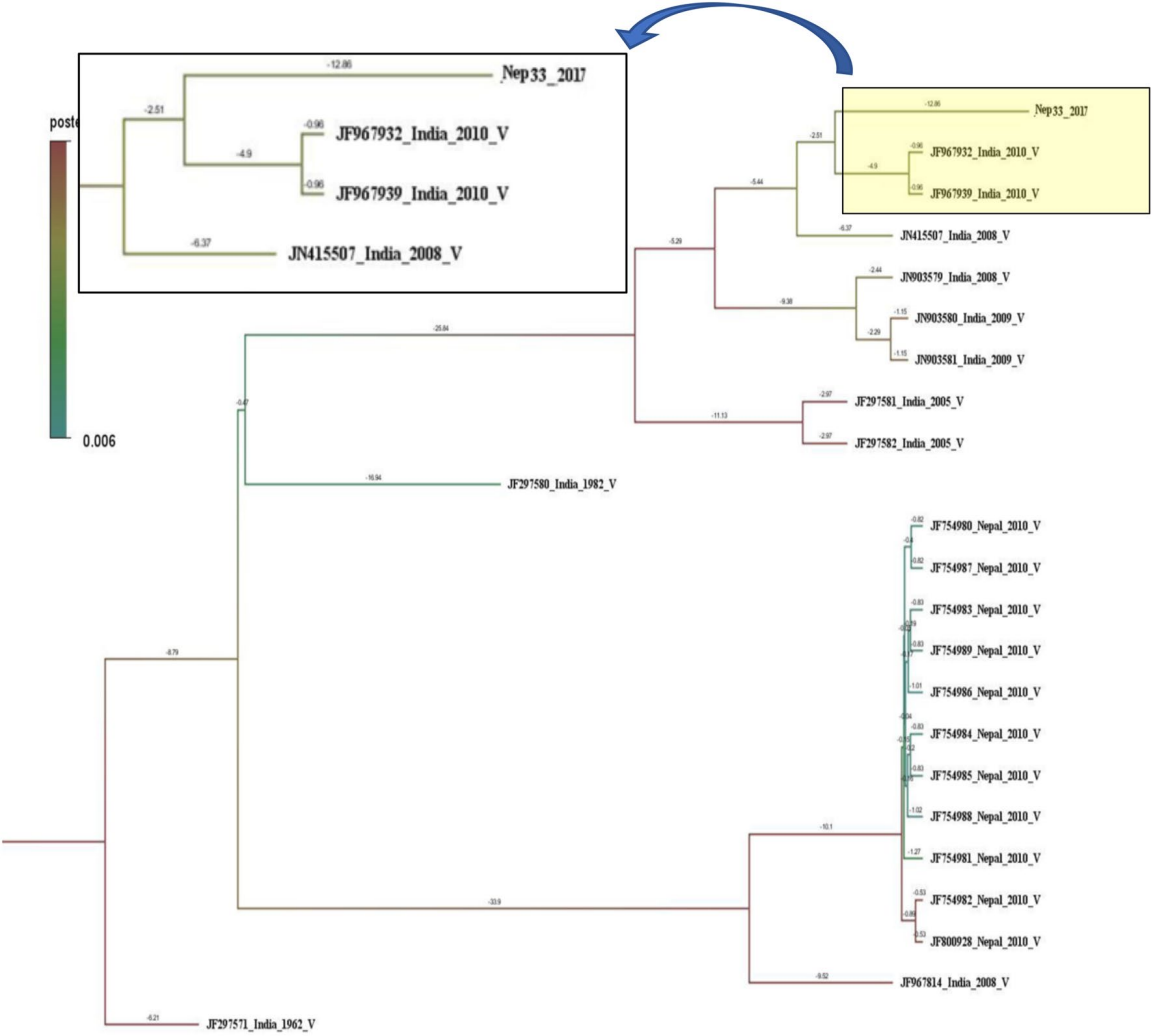
Phylogeny tree of DENV1 serotype constructed using BEAST v2.5.1 using Gamma + I + T93 substitution model against Dengue genotyping database. All the sequences in the tree are labelled as; accession no._country year of isolation genotype. Tree is labelled with posterior probability at node and branch time indicates the length of the respective branch. Branch color is also highlighting posterior probability with a color gradient as indicated in the legends. Detail tree with all of the sequences used for genotyping is shown in Supplementary Figure S2.

Five randomly selected PCR products (one from DENV-1 and four from DENV-2) of the 2017 samples were sequenced for bidirectional sequencing of E-gene and construction of the phylogenetic tree. DENV-1 showed two distinct populations in terms of evolution (Supplementary Figure S2). The DENV-1 reported in this study (Nep33_2017) was found closer to the Indian isolates of genotype V. The isolate was very close to the Indian strains (JF967932_ India_2010_ V and JF967939_ India_2010_ V) which were found split about 5.44 years ago and detected in Nepal in this study after 12.86 years. However, the Nep33_2017 DENV-1 isolate was different from previous strain Nepal_2010_V indicating a diverse population of DENV1 that circulates currently in Nepal (Fig. 2).

**Figure 3:**
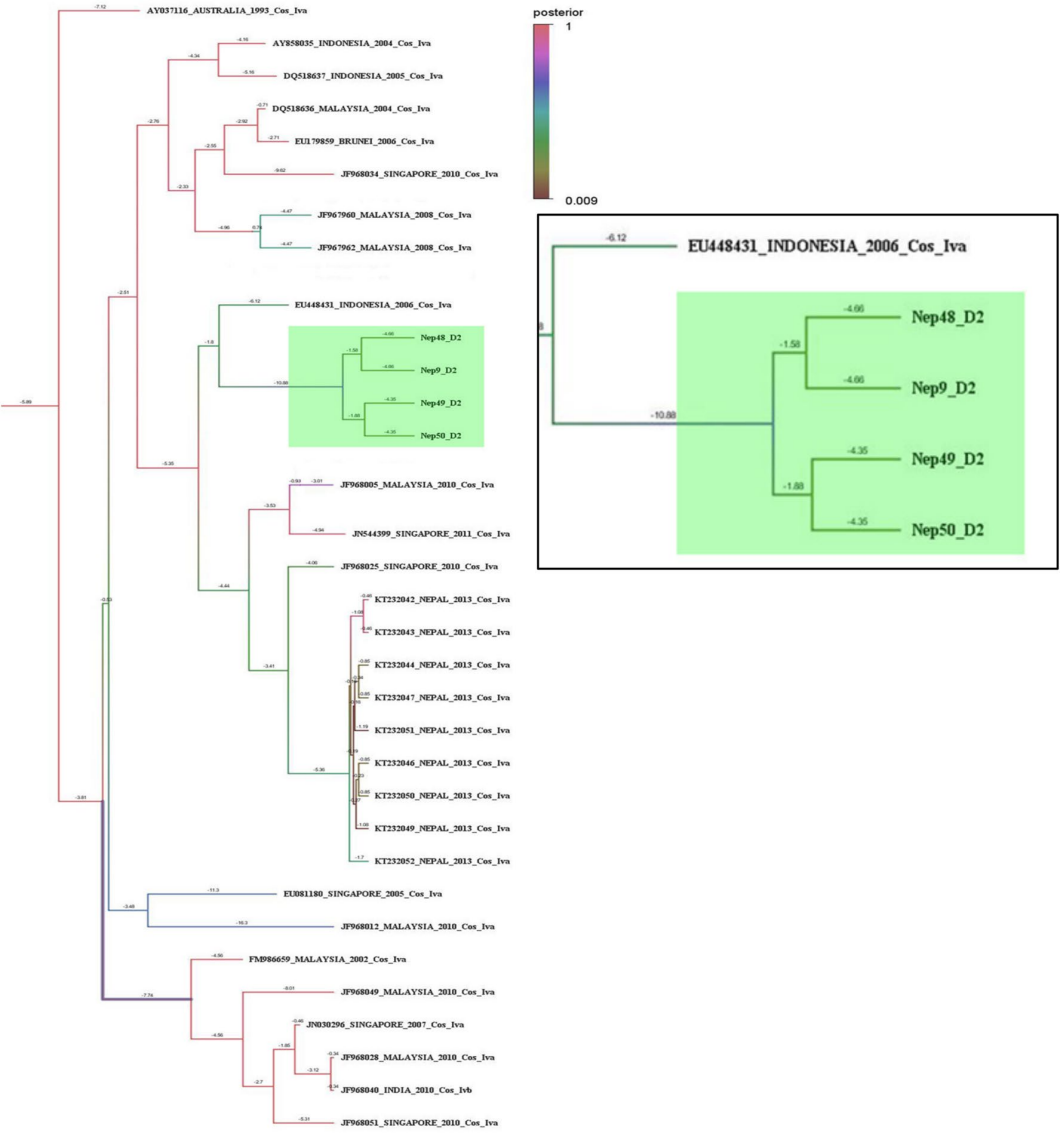
Phylogeny tree of DENV-2 genotype constructed using BEAST v2.5.1 using Gamma + I + T93 substitution model against Dengue genotyping database. All the sequences in the tree are labelled as; accession no._country_year of isolation_genotype. Tree is labelled with posterior probability at node and branch time indicates the length of the respective branch. Branch color is also highlighting posterior probability with a color gradient as indicated in the legends. Detail tree with all of the sequences used for genotyping is shown in Supplementary Figure S3.

DENV-2 detected in four sequenced samples in this study were more similar to Indonesian isolates of genotype cosmopolitan IVa (Fig. 3). Further, the isolates were grouped in different clade than of the Nepal isolate of 2013 cosmopolitan genotype IVa which was split about 18 years earlier. In addition, the next Asian II genotype noted in 2013 was far away from this genotype IVa (Fig. 3, Supplementary Figure S3).

### Expansion of endemicity of dengue from Terai to Hill geographical regions of Nepal

A total of 61 confirmed dengue patients enrolled in this study were from the hospitals; STIDH (n = 30), CMC (n = 14), UCMS (n = 2), BHs (n = 7), GPC (n = 5), NH (1), SH (2) and the patients were inhabitants of plain Terai and Hill region but non from the Himalayan region of Nepal. Prevalence of dengue were detected in all provinces except Karnali Province and covered nine districts each from Terai and Hill region. Comparative analysis of infection trend in Terai showed that there were 68.1% subjects (n = 32/47) from nine districts in the year 2017 while it was 14.3% (n = 2/14) from two districts in 2018. Interestingly, the number of cases in Hill regions increased to 85.7% (n = 12/14) in 2018 while it was only 31.9% (n = 15/47) in 2017. In the fourteen samples studied in 2018, dengue gripped five districts (Kathmandu, Syanja, Ramechhap, Kaski and Nuwakot) in the Hill regions (Table 2). Moreover, more cases were from the Bagmati Province among Hill regions (n = 10/14) which includes Kathmandu, the capital city of Nepal (Supplementary Table S4).

**Table 2:** Geographical distribution of the dengue virus infections, and their serotype and infection patterns in the study period 2017–2018.

Geographical region	Year (No.)	Districts	Province	Serotype	Primary infection	Secondary infection	Sero negative	Total
Terai	2017 (47)	Jhapa, Rautahat, Sarlahi, Chitwan, Nawalpur, Kapilvastu, Rupandehi, Bardia, Kailali	Koshi, Madhesh Pradesh, Bagmati, Gandaki & Sudur Pashchim	DENV1 DENV2 DENV3	16 (34.0%)	11 (23.4%)	5 (10.6%)	32 (68.1%)
	2018 (14)	Rauthat, Chitwan	Madhesh	DENV2	1 (7.1%)	1 (7.1%)	0 (0.0%)	2 (14.3%)
Hill	2017 (47)	Dhading, Nuwakot, , Tanahu, Ramechhap, Kaski, Makawanpur	Bagmati, Lumbini	DENV2	9 (19.1%)	5 (10.6%)	1 (2.1%)	15 (31.9%)
	2018 (14)	Kathmandu, Syangja Ramechhap, Nuwakot, Kaski	Bagmati, Gandaki	DENV2	5 (35.7%)	2 (14.3%)	5 (35.7%)	12 (85.7%)
			Total		31 (50.8%)	19 (34.4%)	10 (16.4%)	61

Previously dengue used to be found only in plain tropical and subtropical Terai regions of Nepal but it showed, endemicity is spreading rapidly towards cold climatic Hill regions too. In our study, the altitude of infected districts ranged from 100 to 7809 m. Although the highest number of cases in the year 2017 i.e., 21.27% (n = 10/47) were seen in Sarlahi District of Province Madhesh Pradesh. The cases were highest50% (n = 7/14) in Kathmandu District of Bagmati Province among Hill regions which has temperate climate (Supplementary Table S4).

### Infection patterns: Primary versus secondary infection

Half of the infected population (50.8%, n = 31/61) were detected as primary infection while 31.5% (n = 19) and 18.0% (n = 11) remained secondary and seronegative respectively. In the year 2017, majority of the confirmed patients visited the hospitals from different districts were of primarily infected (53.2%, n = 25/47) followed by secondarily infected (34.0%, n = 16) and remaining 12.8% (n = 6/47) were seronegative. In the year 2017, only the primary infection (25.13%, n = 10/47) was seen in Kapilvastu, Nawalpur and Bardiya of Terai, and Nuwakot, Tanahun and Chitwan of Hill landscape (Supplementary Table S4). In the year 2018 the primary infection was 42.9% (n = 6/14) followed by 21.4% secondary and 35.7% seronegative. Out of the six primary infections, 83.3% (n = 5) were from Kathmandu, the Hill region. It was the first incidence that dengue disease emerged in the capital city, Kathmandu. The above result of window period explained the expansion of dengue infections to hilly regions.

### Clinical manifestation and age category of dengue patients

The frequency of the most common symptoms in the dengue confirmed patients were fever, and most common were muscles and joint pain and vomiting, nausea, rashes, on the priority order. According to the symptoms observed, 61.70%, 34.04% and 4.25% cases were respectively classified as dengue without warning signs, dengue with warning signs and severe dengue respectively according to WHO case definition of dengue, The total number of dengue virus-infected males were 73.8% (n = 45) and females were 26.2% (n = 16) with male–female ratio 2.8:1. A maximum number of dengue cases were seen in the age group 16–30 followed by 31–45 [Supplementary Figure S5].

### Comparison of clinical parameters on hematology and hepato-enzymes

The descriptive statistics and the hypotheses test for two independent populations’ (dengue and healthy control) means, and simple logistics regression (response Y = 1 for dengue and Y = 0 for healthy) results showed differences in platelet count AST and ALT (Table 3). Shapiro–Wilk normality test conducted to check if variables meet the normality assumption for parametric tests for the healthy control group on the small sample size (n = 15) showed *p*-values larger than 0.05 except for AST. Therefore, platelets, ALT, hematocrit and hemoglobin data for the control healthy group were approximately normal.

**Table 3:** Normality test and the hypothesis test for two independent means.

Biochemical tests	Normality test for control case	Hypothesis test for two independent population			
		Welch t-test			Mann Whitney U test
		t-score	Degree of freedom	*P*-value	*P*-value
Platelets	0.6322	9.92	22.01	0.0000000014	0.0000000264
AST	0.004019	–	–	–	0.0000059570
ALT	0.09607	−4.29	57.63	0.00006907	0.004583
Hematocrit	0.6865	0.33	59.44	0.7393	0.2427
Hemoglobin	0.6247	−0.57	7.21	0.5831	0.6364

The analysis showed that mean and median platelets counts are much less for dengue patients than the healthy group. The mean platelet counts of the dengue patient versus healthy control showed 105,103 (8471 Std. Error) and 288,800 (16,469 Std. Error) respectively (Table 4). The Welch t-test for the platelets count had t-value 9.92 with a very small *p*-value 1.39 × 10^–9^ and the Mann Whitney U test with *p*-value 2.64 ×  × 10^–8^(Table 3). Both of these tests showed significantly lower platelet count in dengue patient than in healthy group. Another possible indicator for the dengue case were AST and ALT which showed the mean and median values for the dengue patients had larger values than the healthy group (Table 4). The means for AST of the control versus dengue case were respectively 25.9 (2.50 Std. Error) and 71.1 (6.80 Std. Error), and for ALT were respectively 36.3 (4.20 Std. Error) and 76.3 (8.30 Std. Error) (Table 4). The Mann Whitney U test for AST had *p*-value 5.96 × 10^–6^. The Welch t-test for ALT has a − 4.29 t-value with *p*-value 0.00006907 and Mann Whitney U test *p*-value was 0.004583. These tests showed significantly different AST and ALT for dengue patients than the healthy group. It was also noted that the platelets test had much smaller *p*-values compared to AST and ALT; which clearly showed that platelets are a much stronger indicator with significant support by AST and ALT to discriminate a dengue patient. The hypothesis tests showed that hematocrit, and hemoglobin were not significantly different between dengue and healthy group, *p*-value larger than the level of significance 0.10 (Table 3, Supplementary Figure S4).

**Table 4:** Summary statistics of platelets, AST, ALT, hematocrit and hemoglobin for healthy versus Dengue patients.

Biochemical tests	Patient status	Median	Mean	Standard deviation	Coefficient of variation	Standard error	Bar plot for mean (margin of error 99%) Green healthy, Red dengue
Platelets	Dengue	96,000	105,103	64,516	0.6	8471	
	Healthy	286,000	288,800	63,783	0.2	16,469	
AST	Dengue	62	71.1	42.8	0.6	6.8	
	Healthy	23	25.9	9.8	0.4	2.5	
ALT	Dengue	58	76.3	55.7	0.7	8.3	
	Healthy	35	36.3	16.4	0.5	4.2	
Hematocrit	Dengue	41	41.4	14.6	0.4	2.1	
	Healthy	42.9	42.2	4	0.1	1	
Hemoglobin	Dengue	14	13.9	1.9	0.1	0.3	
	Healthy	13.2	13.6	1.1	0.1	0.5	

## Discussion

Dengue disease detected for the first time in Nepal from a Japanese traveler in the year 2004 became an emerging infectious disease with the sudden outbreak of 32 cases in the year 2006 and fluctuated around the similar number every year till 2009^21^. Then after, the infection established towards alarming trend having five consecutive outbreaks in every 3 years in 2010, 2013, 2016, 2019 and 2022, and the numbers were exponentially high in the last two outbreaks^22^.

This study carried in the year 2017 and 2018 is focused to the window period between the two outbreaks of 2016 and 2019. The research aimed for the dynamic study on the shift of serotypes, disease expansion to temperate climatic regions, hematologic/hepatic enzymatic makers, primary verses secondary infections, and evolutionary relation of serotypes/genotype of the virus. In this hospital-based cross-sectional study, dengue cases were sampled from 15 (2017) and 7 (2018) districts out of 28 and 43 respectively as reported by EDCD but covered all the provinces except the Karnali Province. The result showed that endemicity of dengue continued to spread towards highland hilly temperate regions since cases were detected in Dhading and Nuwakot districts, the adjoining districts of Kathmandu in the year 2017 drawing a serious concern of expansion to highlands of Nepal. The threat became realistic in the year 2018 when the *Aedes spp.* mosquito transmitted the disease to bunch of people (n = 6) only from a small pocket area named Dhungedhara within a periphery of about 25 households of the densely populated Kathmandu core city for the first time. The major reasons behind are quick transportation facility, rapid urbanization and better adaptation of *Aedes spp.* mosquito towards comparatively cold environment. Now, in this 2022-outbreak, 75 districts have been found infected by dengue^23^. The average infection trend in the study population showed higher primary infections (23.0%, n = 14/61) than the secondary infection (11.5%, n = 7/61) which was also seen in the both years of the window period (19.1% vs. 10.6% in 2017 and 35.7% vs. 14.3% in 2018). Similar trend was prevalent in the terai region with more primary infections (27.9%, n = 17/61) than secondary (19.7%, n = 12/61). Similarly, the average primary infections remained higher (50.8%, n = 31/61) during the study period which is in contrast to the previous studies having dominance of secondary dengue infection^23,24^. The infection pattern indicates more new cases, rapid spreading dengue virus and the low-immune profile of Nepalese population against dengue. This demographic study is suggestive to the concern authorities to be ready for the next cyclic outbreak presumably was expected outbreak 2019 which became true with exponentially increased cases.

The diagnostic approach in Nepal is the cost-inefficient and relied on imported rapid dengue diagnostic kits. Thrombocytopenia is a major parameter of hematologic data as marker for clinical presumption of diagnosis. This study showing significantly low mean platelet count with a very small *p* value (Tables 3 and 4). A negative coefficient of the platelets in logistic regression and odd ratio indicated that an increase in platelets will decrease the probability of being dengue. By increasing thousand-unit platelets count, it is expected 2.72% decrease in the odds of being dengue (Table 5). This study highlighted additional clinical parameters useful for the prognosis and/or diagnosis of dengue. The hypothesis test showed the AST and ALT were also significant supporting the possibility of markers for the disease diagnosis. A positive coefficient of AST and ALT in the logistic regression indicated that an increase in the enzymes will increase in probability to be a dengue patient. One unit increase in AST and ALT will increase an average 10.06% and 3.46% odds of being a dengue patient respectively (Table 5). However, some of the dengue patients in this study had shown low values of AST and/or ALT in this study, which may be due to other reasons than dengue. Hence, this study opened an avenue to work on larger samples for biochemical studies and establish a marker for clinical dengue diagnosis.

**Table 5:** Logistic regression with one explanatory variable.

	Coef.	Std. Err.	z	*P* > z	Odd ratio
Platelets					
Constant	6.509	1.438	4.525	0.00000604	
Platelets	− 2.756E−05	6.603E−06	− 4.173	0.00003	0.9999724
AST					
Constant	− 2.72308	1.04141	− 2.615	0.00893	
AST	0.09589	0.03192	3.004	0.00266	1.1006
ALT					
Constant	− 0.57917	0.68695	− 0.843	0.3992	
ALT	0.03398	0.0152	2.235	0.0254	1.0346

The infection of dengue is not well understood due to antibody-dependent enhancement (ADE), original T cell antigenic sin and viral virulence^25^, although viral serotype shift and genetic drift play major roles. There had always been a dominant serotype either of DENV1 or DENV2 infecting the Nepalese territory. DENV1 was responsible for the 2010 and 2016 while it was DENV2 for the year 2013 outbreak. Interestingly, heterospecific dengue serotype prevalence in the same year is once again observed in the year 2017 after 2006 and 2015^9,24^. However, circulating serotypes in Nepal in the years 2007, 2008, 2009, 2011, and 2012 have not seen reported by government or any institutions.

Though dengue has become an endemic disease of Nepal from the year 2006, only few attempts have been made to identify the genotype and their clades, and whole genome sequencing in only two virus isolated from 2015 dengue infected subjects^26^. The reports of genotype were IVa of DENV2 in 2004 infection referred as genotype 1 by Takasaki et al., 2008^8^, genotype V of DENV1 in 2010^10^ and, genotype Cosmopolitan IVb and Asian II in 2013^11^ In this study (Nep 33_2017), the DENV1 genotype V is though found close to the genotype V of Nepal seen in the year 2010^10^, it showed a separate lineage which was split around 40 years ago. So, this genotype grouped in the separate clade from the previous one being closer to 2010 Indian strain of genotype V (Fig. 2). Unlike to dengue serotype 1, DENV-2 genotype (Nep_9/48/49/50_D2) of this study is not closer to Indian genotype IVb, however, showed closeness with genotype IVa of Indonesian and other East Asian strains which indicated DENV-2 circulating in Nepal have a different origin. So, the circulating DENV-2 is of Cosmopolitan genotype IVa, somewhere close to the genotype circulating in the year 2013 epidemic^11^ which were separated around 14 years back. The result obtained in this study targeting envelope region was very similar to our previous phylogeny work on envelope region of dengue with different set of primers ^27^. The previously reported Asian II genotype of dengue serotype 2 from 2013, however, was similar to USA strain^27^ and the detection in Nepalese territory could be due to accidental spillover probably by tourists. Because of such co-circulation of three serotypes of dengue, the sudden resurgence of severe dengue disease can occur in near future due to antibody-dependent enhancement (ADE)^4,28^.

Our study found that DENV-1 and DENV-3 were found to be circulating in the Terai lower belt whereas DENV-2 was noted as virulent genotype acclimatizing to the hilly district such as Kathmandu, Dhading and Nuwakot. Circulation of DENV-2 in hilly regions which was previously thought as safe has been ventured by Genotype Cosmopolitan IVa unlike cosmopolitan IVb found in India, which is closely related to South-East Asia (Malaysia, Singapore). It indicates an unusual spillover of DENV-2 in Nepal. Increased average global temperature by 0.94 °C till 2016 could also be reason behind the dengue spreading to higher altitude^29^ and partly could be due to this unusual venture of Genotype Cosmopolitan IVa. DENV-1 was close to Indian genotype (Genotype V) and is limited to Terai region as expected. Genotypes from different parts of the world if reach to previously considered non-endemic region, there is chance of quick spread to a point towards potential outbreak.

In reference to the DENV-3, the serotype has been seen in the year 2015, and 2017 has never been a dominant one but can be considered as in incubation with future risk of progressing to virulent based on the role of ADE. This study has revealed the Nepal specific dengue serotype and genotype which is very essential for the understanding of epidemiological status of dengue infection. Further, the information on hematological parameters of dengue would be useful as presumptive marker during the unavailability of rapid diagnostic kit.

## Methods

### Ethics information

The study was approved by the Nepal Health Research Council (NHRC) (Reg. no. 378/2016) which has the authority to approve the study in Nepal. An approved version of informed consent forms was used for participation and collection of biological specimens. All adult subjects provided written informed consent, and a parent or guardian of any child participant provided written informed consent on their behalf. All the methods were performed in accordance with approved NHRC guidelines and regulations.

### Design

A cross-sectional descriptive study on 61 dengue cases from different hospitals in Nepal was carried out.

### Sampling plan

The samples of 2017 were collected from Sukraraj Tropical and Infectious disease Hospital (STIDH)—Kathmandu, Chitwan Medical College and Teaching Hospital (CMC)-Chitwan, Universal College of Medical Science (UCMS)-Bhairahawa and Hospitals in Butwal (BHs)—Lumbini Zonal Hospital, Butwal Hospital and National Path Lab while the 2018 samples were from STIDH, Norvic International Hospital (NH), Suvekchya International Hospital and Research Center (SH) and Gorakh Kali Polyclinic (GPC) from Kathmandu. The samples in both the years were collected in the 3 months’ time duration from September to November, which were the peak months for dengue epidemics in the year 2017 and 2018.

Demographic information, symptoms and diagnosis were recorded by attending physicians. Patients with an acute febrile illness of 2–7 days duration and showing the symptoms of dengue fever (fever with two of the following symptoms: nausea, vomiting, rashes, aches and pains, leukopenia and positive Tourniquet test) were enrolled in the study. WHO, 2009 guidelines were strictly followed for determination of dengue-case identification^15^. Samples were not collected from HIV patients, children < 5 years, pregnant women and subjects denying to sign the informed consent form (ICF) at the time of sample collection.

### Laboratory validation of dengue cases and subject enrollments

The suspected dengue samples from the year 2017 and 2018 were tested by ELISA (NS1 antigen, and anti-DENV IgM and IgG antibody detection) and Polymerase Chain Reaction (Real-Time Reverse Transcriptase PCR and Nested PCR for viral nucleic acid detection). The samples positive to either NS1, IgM, and/or PCR were classified as confirmed dengue cases (n = 61). Biochemical parameters, like platelets count, hematocrit and hemoglobin, aspartate transaminase (AST), and alanine transaminase (ALT) were performed. Volunteers who did not show any signs of dengue or other illness (n = 15) were enrolled as healthy controls.

### Serological tests

ELISA was carried for detection of anti-dengue IgG and IgM antibodies and dengue antigen (NS1) using the standard InBios kit (InBios International Inc., Seattle, WA, catalogue no. DDGS-R for IgG, DDMS-1 for IgM and DNS1-R for NS1). The tests were performed in duplicates and readings were taken at 450 nm wavelength in ELISA Plate reader (Thermo Multiskan EX) and Immune Status Ratio (ISR) values were calculated to define whether the samples were dengue cases or not as described in the kit manuals.

The ratio of anti-dengue IgM to IgG ≥ 1.2 was grouped in primary infection while less in secondary infection. The ratio was confirmed as valid for the sera samples diluted in the ratio 1:100^17^. The samples with ISR negative to both anti-dengue IgG and IgM were termed as seronegative.

### Molecular assays

Dengue patient serum samples (140 uL) were used for viral RNA extraction by using QIAamp® Viral RNA Mini kit using spin protocol (Cat. No. 52904). cDNA was prepared using ProtoScript® First Strand cDNA Synthesis Kit (Cat. No. E6300S) from the dengue RNA as per manufacturer’s instruction for E gene based Nested PCR assay. Molecular diagnosis and serotyping of dengue were carried by nested PCR and Real Time RT-PCR assay (Multiplex) following the protocol adopted by Prajapati et al. (2020) with minor modifications.

### DENV serotype analysis by real-time polymerase chain reaction

Reverse Transcriptase Real-Time PCR was performed (BIORAD-CFX96 Touch™ Real-Time PCR Detection System) using the master mix prepared with primers, probes and superscript III enzyme for tests (n = 61) and controls (n = 15) according to the instruction on Center for Disease Control and Prevention (CDC), USA guideline for identification of DENV serotypes. The results were interpreted as positive to DENV-1, 2, 3 and 4 if the amplification of probes; FAM (Blue), VIC (Green), Texas Red (Red) and Cy5 (Purple) curve were amplified respectively within CT value 37.

### Primer design

Primers used in this study were designed on the basis of the gene sequence uploaded to GenBank NCBI. The non-overlapping and non-redundant sequences with appropriate melting point were designed using the Benchling software (SA, USA). The accuracy of the primer was calculated by nBLAST (NCBI), and sequences with more than 99.9% alignment for given serotype was included in the study (Supplementary Table S1).

### Nested RT-PCR

Nested PCR of the cDNA was performed for all the Real Time RT-PCR positive samples using the envelope protein primers (Supplementary Table S1) and Solis Biodyne -5 × FIREPol® Master Mix (Cat. No. 04-11-00,125) according to Prajapati et al. (2020) with minor modification. Primers AA6EP_F (10 pm/µl) and AA7EP_R (10 pm/µl) were used for the first round PCR. The PCR conditions were set to initial denaturation at 98 °C for 5 min followed by 35 cycles of the PCR with cycling conditions of denaturation 95 °C, annealing 59 °C and extension 72 °C. The final extension was done at 72 °C for 5 min and the reaction was kept at hold at 4 °C. The PCR product was diluted in the ratio 1:10 and used as template for second round PCR using serotype-specific primers (AA8EP_F & AA9EP_R for DENV1, AA10EP_F & AA11EP_R for DENV2, AA12EP_F & AA13EP_R for DENV3 and AA14EP_F & AA15EP_R for DENV4). The PCR conditions were same as of the first round and only annealing temperature was different for different serotypes (54.5 °C, 53.5 °C, 56 °C and 53.5 °C for DENV-1, DENV-2, DENV-3 and DENV-4 respectively). Agarose gel electrophoresis was run in 1.5% agarose and the bands were visualized under UV transilluminator.

### Sequencing and genotyping by phylogeny

Five randomly selected PCR products (one from DENV-1 and four from DENV-2) of the 2017 samples were sent to Xcelris Pvt Ltd., India for bidirectional sequencing. The obtained sequences were processed by using software FinchTV v1.4.0 & Bioedit v7.0.5.3 and were visualized in Aliview v1.23. Genotyping of the sequences were performed by phylogeny method against dengue virus genotyping database^18^. There were 82 and 149 sequences for DENV-1 and DENV-2 respectively which were tallied to get the phylogeny tree (Supplementary Tables S2 & S3). The gene sequences of the envelope protein obtained in this study were deposited in GenBank with accession numbers MK209641-MK209645 (Supplementary Table S2 & S3).

Phylogenetic analyses were carried and phylogenetic tree was constructed using the time to a most recent common ancestor (TMRCA) with an average clock rate of 10^–3^ under GTR + I + T93 substitution model^19^. A Bayesian skyline coalescent distribution was implied. Markov Chain Monte Carlo (MCMC) were run for 40–50 million generation to get adequate RSS (Random Sample Size) value of > 100 in BEAST v2.5.1. Dengue genotype database were used for a separate phylogenetic tree construction with tip dates for DENV1 and DENV2^18^ along with previously reported sequences from Nepal. The runs were visualized for quality and summarized by Tracer v1.7.1 and Tree annotator v2.5.1 respectively. Figtree v1.4.3 was the software used for visualization and edit of all trees^20^.

### Statistical analysis

All the data obtained in this study was recorded in MS-Excel 2016. The descriptive statistics, demographic, geographic, time variables and clinical parameters were analyzed. The hematological and hepatoenzymatic study had a total of 76 cases (dengue patients-61 and healthy control-15). The variables: platelets, AST, ALT, hematocrit, and hemoglobin were used in this study. For each variable the summary statistics: median, mean, standard deviation, coefficient of variance and standard error for mean were generated.

The parametric and non-parametric hypothesis tests were used to study if the two independent samples from dengue and healthy groups have the same central value. Shapiro–Wilk normality test were conducted to check if variables meet the normality assumption for parametric tests. The simple explanatory logistic regression model was used to study the probability and odds for dengue cases compared to healthy groups.

### Ethics

For each patient who had given informed consent, a questionnaire consisting of demographic and clinical features were enrolled in the study according to the ethical approval from Nepal Health Research Council (NHRC) (Reg. no. 378/2016).

## Supplementary Information

Supplementary Information.

## Data Availability

The gene sequences of the envelope protein obtained in this study were deposited in GenBank with accession numbers MK209641-MK209645 (Supplementary Tables S2 & S3).
