# Supplementary material for: Dengue virus infection during window period of consecutive outbreaks in Nepal and assessment of clinical parameters
Source: Sci Rep. 2023 Jun 7;13:9262. doi: 10.1038/s41598-023-35928-5 (PMC10247790; doi:10.1038/s41598-023-35928-5)
Supplement: Supplementary file 1 — Supplementary Information. [file 41598_2023_35928_MOESM1_ESM.pdf]

Supplementary tables and figures.

Supplementary Table S1: Primers used for dengue virus confirmation and serotype specific PCR

Primer	Sequence	Location (w.r.t. ref. seq.)	Ref. seq. (GenBank accession no.)	Expected size (bp)
AA6EP_F	TGGCTGGTGCACAGACAATGGTT	616-638	MF381049.1	600
AA7EP_R	GCTGTGTCACCCAGAGTGGCCAT	2146-2168	KY849753.1	
AA8EP_F	GGGGCTTCAACATCCCAAGAG	667-687	MH680237.1	500
AA9EP_R	GCTTAGTTTCAAAGCTTTTTCAC	1170-1148	MG933845.1	
AA10EP_F	ATCCAGATGTCATCAGGAAAC	808-828	MH110734.1	337
AA11EP_R	CCGGCTCTACTCCTATGATG	1153-1134	MG895167.1	
AA12EP_F	CAATGTGCTTGAATACCTTTGT	893-914	MG895205.1	189
AA13EP_R	GGACAGGCTCCTCCTTCTTG	1089-1071	MH173166.1	
AA14EP_F	GGACAACAGTGGTGAAAGTCA	953-973	MH178419.1	138
AA15EP_R	GGTTACACTGTTGGTATTCTCA	1095-1074	MG895393.1	

Details of primers sharing more than 99.9% used for the serotype segregation of dengue virus obtained from nucleotide blast in NCBI.

Supplementary Table S2: Sequences used in phylogeny of DENV1 along with all details.

Accession no.	Country	year	Genotype	Name in phylogeny
AB111071	Cambodia	2001	I	AB111071_Cambodia_2001_I
AB597966	Indonesia	2010	I	AB597966_Indonesia_2010_I
AB597967	Indonesia	2010	I	AB597967_Indonesia_2010_I
AF298808	Djibouti	1998	I	AF298808_Djibouti_1998_I
AF309641	Cambodia	1998	I	AF309641_Cambodia_1998_I
AF425610	Angola	1988	V	AF425610_Angola_1988_V
AM746212	Saudi Arabia	2006	I	AM746212_SaudiArabia_2006_I
AM746213	Saudi Arabia	2006	I	AM746213_SaudiArabia_2006_I
AM746215	Saudi Arabia	2005	I	AM746215_SaudiArabia_2005_I
AY732390	Thailand	1983	I	AY732390_Thailand_1983_I
AY732397	Thailand	1982	I	AY732397_Thailand_1982_I
AY732481	Thailand	1982	I	AY732481_Thailand_1982_I
DQ265138	Malaysia	2002	I	DQ265138_Malaysia_2002_I
DQ265157	Myanmar	2002	I	DQ265157_Myanmar_2002_I
DQ285558	Reunion	2004	IV	DQ285558_Reunion_2004_IV
DQ285561	Seychelles	2004	IV	DQ285561_Seychelles_2004_IV
EU069598	Singapore	2003	I	EU069598_Singapore_2003_I
EU069599	Singapore	2002	I	EU069599_Singapore_2002_I
EU069600	Singapore	2002	I	EU069600_Singapore_2002_I
EU069601	Singapore	2003	I	EU069601_Singapore_2003_I
EU081279	Singapore	2005	I	EU081279_Singapore_2005_I
EU081281	Singapore	2006	I	EU081281_Singapore_2006_I
FJ639669	Cambodia	2000	I	FJ639669_Cambodia_2000_I
FJ639670	Cambodia	2001	I	FJ639670_Cambodia_2001_I
FJ639671	Cambodia	2001	I	FJ639671_Cambodia_2001_I
FJ639672	Cambodia	2001	I	FJ639672_Cambodia_2001_I
FJ639673	Cambodia	2001	I	FJ639673_Cambodia_2001_I
FJ639674	Cambodia	2002	I	FJ639674_Cambodia_2002_I
FJ639675	Cambodia	2003	I	FJ639675_Cambodia_2003_I
FJ639676	Cambodia	2003	I	FJ639676_Cambodia_2003_I
FJ639677	Cambodia	2003	I	FJ639677_Cambodia_2003_I
FJ639678	Cambodia	2003	I	FJ639678_Cambodia_2003_I
FJ639679	Cambodia	2003	I	FJ639679_Cambodia_2003_I
FJ639680	Cambodia	2003	I	FJ639680_Cambodia_2003_I
FJ639681	Cambodia	2003	I	FJ639681_Cambodia_2003_I
FJ639682	Cambodia	2004	I	FJ639682_Cambodia_2004_I
FJ639683	Cambodia	2005	I	FJ639683_Cambodia_2005_I
FJ639684	Cambodia	2005	I	FJ639684_Cambodia_2005_I
FJ639685	Cambodia	2005	I	FJ639685_Cambodia_2005_I
FJ639686	Cambodia	2006	I	FJ639686_Cambodia_2006_I
FJ639687	Cambodia	2006	I	FJ639687_Cambodia_2006_I
FJ744702	Cambodia	2006	I	FJ744702_Cambodia_2006_I

FJ850069	Cambodia	2003	I	FJ850069_Cambodia_2003_I
FJ882559	Vietnam	2007	I	FJ882559_VietNam_2007_I
FJ882560	Vietnam	2007	I	FJ882560_VietNam_2007_I
FR666922	Malaysia	2004	I	FR666922_Malaysia_2004_I
GQ199782	Vietnam	2007	I	GQ199782_VietNam_2007_I
GQ199783	Vietnam	2007	I	GQ199783_VietNam_2007_I
GQ357666	Singapore	2008	I	GQ357666_Singapore_2008_I
GQ357689	Singapore	2007	I	GQ357689_Singapore_2007_I
GQ868602	Philippines	2004	IV	GQ868602_Philippines_2004_IV
GQ868618	Cambodia	2003	I	GQ868618_Cambodia_2003_I
GQ868637	Cambodia	2000	I	GQ868637_Cambodia_2000_I
GU131923	Cambodia	2005	I	GU131923_Cambodia_2005_I
GU131965	Mexico	2007	V	GU131965_Mexico_2007_V
HM181949	Cambodia	2007	I	HM181949_Cambodia_2007_I
JF297571	India	1962	V	JF297571_India_1962_V
JF297572	India	1962	V	JF297572_India_1962_V
JF297576	India	1963	V	JF297576_India_1963_V
JF297577	India	1963	V	JF297577_India_1963_V
JF297580	India	1982	V	JF297580_India_1982_V
JF297581	India	2005	V	JF297581_India_2005_V
JF297582	India	2005	V	JF297582_India_2005_V
JF297583	India	2005	V	JF297583_India_2005_V
JF967798	Indonesia	2008	I	JF967798_Indonesia_2008_I
JF967810	Myanmar	2008	I	JF967810_Myanmar_2008_I
JF967814	India	2008	V	JF967814_India_2008_V
JF967868	Indonesia	2009	I	JF967868_Indonesia_2009_I
JF967932	India	2010	V	JF967932_India_2010_V
JF967939	India	2010	V	JF967939_India_2010_V
JN029811	China	2010	I	JN029811_China_2010_I
JN029812	China	2010	I	JN029812_China_2010_I
JN415507	India	2008	V	JN415507_India_2008_V
JN415515	Palau	2000	IV	JN415515_Palau_2000_IV
JN415531	Australia	2008	I	JN415531_Australia_2008_I
JN819423	Cambodia	2001	I	JN819423_Cambodia_2001_I
JN903578	India	2007	V	JN903578_India_2007_V
JN903579	India	2008	V	JN903579_India_2008_V
JN903580	India	2009	V	JN903580_India_2009_V
JN903581	India	2009	V	JN903581_India_2009_V
JQ287664	Cambodia	2004	I	JQ287664_Cambodia_2004_I
JQ896294	Ireland	2010	I	JQ896294_Ireland_2010_I
MK209645	Nepal	2017		Nep33_2017

Supplementary Table S3: Sequences used in phylogeny of DENV-2 along with all details

Gene Bank Accession No.	Country	Year	Genotype	Name in the Phylogeny
AB111449	India	1996	Cosmopolitan_Genotype_Ivb	AB111449_INDIA_1996_Cos_Ivb
AB111454	Japan	2001	Cosmopolitan_Genotype_Iva	AB111454_JAPAN_2001_Cos_Iva
AB189122	Indonesia	1998	Cosmopolitan_Genotype_Iva	AB189122_INDONESIA_1998_Cos_Iva
AB194882	Thailand	2004	Asian_I	AB194882_THAILAND_2004_Asian_I
AB194883	Srilanka	2004	Cosmopolitan_Genotype_Ivb	AB194883_SRILANKA_2004_Cos_Ivb
AB194884	Philippines	2004	Cosmopolitan_Genotype_Iva	AB194884_PHILIPPINES_2004_Cos_Iva
AB194885	Nepal	2004	Cosmopolitan_Genotype_Ivb	AB194885_NEPAL_2004_Cos_Ivb
AF004019	Australia	1996	Cosmopolitan_Genotype_Iva	AF004019_AUSTRALIA_1996_Cos_Iva
AF100469	Mexico	1992	Asian_American	AF100469_MEXICO_1992_Asian_American
AF204177	China	1989	Asian_II	AF204177_CHINA_1989_Asian_II
AF204178	China	1987	Asian_II	AF204178_CHINA_1987_Asian_II
AF231720	Thailand	1993	Sylvatic	AF231720_THAILAND_1993_Sylvatic
AF295694	Philippines	2000	Asian_II	AF295694_PHILIPPINES_2000_Asian_II
AF295697	Philippines	2000	Asian_II	AF295697_PHILIPPINES_2000_Asian_II
AF359579	China	1999	Cosmopolitan_Genotype_Ivb	AF359579_CHINA_1999_Cos_Ivb
AF410371	India	1991	Cosmopolitan_Genotype_Ivb	AF410371_INDIA_1991_Cos_Ivb
AF410372	Srilanka	1994	Cosmopolitan_Genotype_Ivb	AF410372_SRILANKA_1994_Cos_Ivb
AF410373	India	1991	Cosmopolitan_Genotype_Ivb	AF410373_INDIA_1991_Cos_Ivb
AF410374	India	1994	Cosmopolitan_Genotype_Ivb	AF410374_INDIA_1994_Cos_Ivb
AF410377	Thailand	1998	Cosmopolitan_Genotype_Iva	AF410377_THAILAND_1998_Cos_Iva
AF410379	Singapore	1991	Cosmopolitan_Genotype_Ivb	AF410379_SINGAPORE_1991_Cos_Ivb
AM746221	Saudi Arabia	2004	Cosmopolitan_Genotype_Ivb	AM746221_SAUDIARABIA_2004_Cos_Ivb
AM746225	Saudi Arabia	1994	Cosmopolitan_Genotype_Ivb	AM746225_SAUDIARABIA_1994_Cos_Ivb
AM746227	Saudi Arabia	1994	Cosmopolitan_Genotype_Ivb	AM746227_SAUDIARABIA_1994_Cos_Ivb
AY037116	Australia	1993	Cosmopolitan_Genotype_Iva	AY037116_AUSTRALIA_1993_Cos_Iva
AY702040	Colombia	1986	Asian_American	AY702040_COLOMBIA_1986_Asian_American
AY706007	Philippines	2003	Cosmopolitan_Genotype_Iva	AY706007_PHILIPPINES_2003_Cos_Iva
AY858035	Indonesia	2004	Cosmopolitan_Genotype_Iva	AY858035_INDONESIA_2004_Cos_Iva
AY512569	Philippines	1995	Asian_II	AY512569_PHILIPPINES_1995_Asian_II
DQ181804	Thailand	1984	Asian_I	DQ181804_THAILAND_1984_Asian_I
DQ364554	Puerto Rico	1988	Asian_I	DQ364554_PUERTO RICO_1988_Asian_I
DQ448231	India	2001	Cosmopolitan_Genotype_Ivb	DQ448231_INDIA_2001_Cos_Ivb
DQ448234	India	2001	Cosmopolitan_Genotype_Ivb	DQ448234_INDIA_2001_Cos_Ivb
DQ448236	India	2001	Cosmopolitan_Genotype_Ivb	DQ448236_INDIA_2001_Cos_Ivb
DQ518633	Philippines	2005	Cosmopolitan_Genotype_Iva	DQ518633_PHILIPPINES_2005_Cos_Iva
DQ518636	Malaysia	2004	Cosmopolitan_Genotype_Iva	DQ518636_MALAYSIA_2004_Cos_Iva
DQ518637	Indonesia	2005	Cosmopolitan_Genotype_Iva	DQ518637_INDONESIA_2005_Cos_Iva
DQ518641	Vietnam	2003	Asian_I	DQ518641_VIETNAM_2003_Asian_I

DQ518646	Thailand	2004	Asian_I	DQ518646_THAILAND_2004_Asian_I
DQ518649	Cambodia	2003	Asian_I	DQ518649_CAMBODIA_2003_Asian_I
DQ518650	Vietnam	2004	Asian_I	DQ518650_VIETNAM_2004_Asian_I
EF105382	Burkina Faso	1980	Sylvatic	EF105382_BURKINAFASO_1980_Sylvatic
EU081180	Singapore	2005	Cosmopolitan_Genotype_Iva	EU081180_SINGAPORE_2005_Cos_Iva
EU179859	Brunei	2006	Cosmopolitan_Genotype_Iva	EU179859_BRUNEI_2006_Cos_Iva
EU448417	Vietnam	2007	Asian_I	EU448417_VIETNAM_2007_Asian_I
EU448418	Philippines	2003	Asian_II	EU448418_PHILIPPINES_2003_Asian_II
EU448420	Vietnam	2006	Asian_I	EU448420_VIETNAM_2006_Asian_I
EU448421	India	2006	Cosmopolitan_Genotype_Ivb	EU448421_INDIA_2006_Cos_Ivb
EU448422	India	2004	Cosmopolitan_Genotype_Ivb	EU448422_INDIA_2004_Cos_Ivb
EU448423	Bangladesh	2005	Cosmopolitan_Genotype_Ivb	EU448423_BANGLADESH_2005_Cos_Ivb
EU448424	Bangladesh	2004	Cosmopolitan_Genotype_Ivb	EU448424_BANGLADESH_2004_Cos_Ivb
EU448425	Indonesia	2007	Cosmopolitan_Genotype_Iva	EU448425_INDONESIA_2007_Cos_Iva
EU448431	Indonesia	2006	Cosmopolitan_Genotype_Iva	EU448431_INDONESIA_2006_Cos_Iva
EU854293	Colombia	1944	Asian_II	EU854293_COLOMBIA_1944_Asian_II
FJ538905	India	2004	Cosmopolitan_Genotype_Ivb	FJ538905_INDIA_2004_Cos_Ivb
FJ538911	India	1993	Cosmopolitan_Genotype_Ivb	FJ538911_INDIA_1993_Cos_Ivb
FJ538912	India	1995	Cosmopolitan_Genotype_Ivb	FJ538912_INDIA_1995_Cos_Ivb
FJ538913	India	1997	Cosmopolitan_Genotype_Ivb	FJ538913_INDIA_1997_Cos_Ivb
FJ538920	India	1974	Cosmopolitan_Genotype_Ivb	FJ538920_INDIA_1974_Cos_Ivb
FJ538922	India	1983	Cosmopolitan_Genotype_Ivb	FJ538922_INDIA_1983_Cos_Ivb
FJ538923	India	1991	Cosmopolitan_Genotype_Ivb	FJ538923_INDIA_1991_Cos_Ivb
FJ538924	India	1990	Cosmopolitan_Genotype_Ivb	FJ538924_INDIA_1990_Cos_Ivb
FJ538925	India	1992	Cosmopolitan_Genotype_Ivb	FJ538925_INDIA_1992_Cos_Ivb
FJ807632	India	2004	Cosmopolitan_Genotype_Ivb	FJ807632_INDIA_2004_Cos_Ivb
FJ807633	India	2005	Cosmopolitan_Genotype_Ivb	FJ807633_INDIA_2005_Cos_Ivb
FJ807634	India	1994	Cosmopolitan_Genotype_Ivb	FJ807634_INDIA_1994_Cos_Ivb
FJ807637	India	1996	Cosmopolitan_Genotype_Ivb	FJ807637_INDIA_1996_Cos_Ivb
FJ807638	India	1996	Cosmopolitan_Genotype_Ivb	FJ807638_INDIA_1996_Cos_Ivb
FJ807639	India	1993	Cosmopolitan_Genotype_Ivb	FJ807639_INDIA_1993_Cos_Ivb
FJ807640	India	1994	Cosmopolitan_Genotype_Ivb	FJ807640_INDIA_1994_Cos_Ivb
FJ898454	India	2006	Cosmopolitan_Genotype_Ivb	FJ898454_INDIA_2006_Cos_Ivb
FJ906959	Papua New Guinea	2008	Asian_II	FJ906959_PAPUA NEW GUINEA_2008_Asian_II
FJ906968	USA	2009	Asian_II	FJ906968_USA_2009_Asian_II
FM986654	Malaysia	1997	Cosmopolitan_Genotype_Iva	FM986654_MALAYSIA_1997_Cos_Iva
FM986659	Malaysia	2002	Cosmopolitan_Genotype_Iva	FM986659_MALAYSIA_2002_Cos_Iva
GQ199901	USA	2008	Asian_II	GQ199901_USA_2008_Asian_II
GQ252677	Srilanka	2004	Cosmopolitan_Genotype_Ivb	GQ252677_SRILANKA_2004_Cos_Ivb
GQ398268	Indonesia	1975	Asian_II	GQ398268_INDONESIA_1975_Asian_II
GU211764	Vietnam	2006	Asian_I	GU211764_VIETNAM_2006_Asian_I
HQ012538	Brazil	1990	Asian_I	HQ012538_BRAZIL_1990_Asian_I
HQ891023	Taiwan	2008	Asian_II	HQ891023_TAIWAN_2008_Asian_II

JN030330	Singapore	2010	Cosmopolitan_Genotype_Ivb	JN030330_SINGAPORE_2010_Cos_Ivb
JF730053	USA	2006	Asian_II	JF730053_USA_2006_Asian_II
JF804033	India	2006	Cosmopolitan_Genotype_Ivb	JF804033_INDIA_2006_Cos_Ivb
JF967960	Malaysia	2008	Cosmopolitan_Genotype_Iva	JF967960_MALAYSIA_2008_Cos_Iva
JF967962	Malaysia	2008	Cosmopolitan_Genotype_Iva	JF967962_MALAYSIA_2008_Cos_Iva
JF967965	Malaysia	2008	Cosmopolitan_Genotype_Iva	JF967965_MALAYSIA_2008_Cos_Iva
JF967991	Phillippines	2009	Cosmopolitan_Genotype_Iva	JF967991_Phillippines_2009_Cos_Iva
JF968005	Malaysia	2010	Cosmopolitan_Genotype_Iva	JF968005_MALAYSIA_2010_Cos_Iva
JF968012	Malaysia	2010	Cosmopolitan_Genotype_Iva	JF968012_MALAYSIA_2010_Cos_Iva
JF968025	Singapore	2010	Cosmopolitan_Genotype_Iva	JF968025_SINGAPORE_2010_Cos_Iva
JF968028	Malaysia	2010	Cosmopolitan_Genotype_Iva	JF968028_MALAYSIA_2010_Cos_Iva
JF968034	Singapore	2010	Cosmopolitan_Genotype_Iva	JF968034_SINGAPORE_2010_Cos_Iva
JF968035	Malaysia	2010	Cosmopolitan_Genotype_Iva	JF968035_MALAYSIA_2010_Cos_Iva
JF968039	Philippines	2010	Cosmopolitan_Genotype_Iva	JF968039_PHILIPPINES_2010_Cos_Iva
JF968040	India	2010	Cosmopolitan_Genotype_Ivb	JF968040_INDIA_2010_Cos_Ivb
JF968049	Malaysia	2010	Cosmopolitan_Genotype_Iva	JF968049_MALAYSIA_2010_Cos_Iva
JF968051	Singapore	2010	Cosmopolitan_Genotype_Iva	JF968051_SINGAPORE_2010_Cos_Iva
JN030296	Singapore	2007	Cosmopolitan_Genotype_Iva	JN030296_SINGAPORE_2007_Cos_Iva
JN030298	Singapore	2010	Cosmopolitan_Genotype_Iva	JN030298_SINGAPORE_2010_Cos_Iva
JN030328	Singapore	2010	Cosmopolitan_Genotype_Ivb	JN030328_SINGAPORE_2010_Cos_Ivb
JN030329	Singapore	2010	Cosmopolitan_Genotype_Ivb	JN030329_SINGAPORE_2010_Cos_Ivb
JN030331	Singapore	2010	Cosmopolitan_Genotype_Ivb	JN030331_SINGAPORE_2010_Cos_Ivb
JN030332	Singapore	2010	Cosmopolitan_Genotype_Ivb	JN030332_SINGAPORE_2010_Cos_Ivb
JN030333	Singapore	2010	Cosmopolitan_Genotype_Ivb	JN030333_SINGAPORE_2010_Cos_Ivb
JN030334	Singapore	2010	Cosmopolitan_Genotype_Ivb	JN030334_SINGAPORE_2010_Cos_Ivb
JN030335	Singapore	2010	Cosmopolitan_Genotype_Ivb	JN030335_SINGAPORE_2010_Cos_Ivb
JN030336	Singapore	2010	Cosmopolitan_Genotype_Ivb	JN030336_SINGAPORE_2010_Cos_Ivb
JN030337	Singapore	2010	Cosmopolitan_Genotype_Ivb	JN030337_SINGAPORE_2010_Cos_Ivb
JN030338	Singapore	2010	Cosmopolitan_Genotype_Ivb	JN030338_SINGAPORE_2010_Cos_Ivb
JN030339	Singapore	2010	Cosmopolitan_Genotype_Ivb	JN030339_SINGAPORE_2010_Cos_Ivb
JN030340	Singapore	2010	Cosmopolitan_Genotype_Ivb	JN030340_SINGAPORE_2010_Cos_Ivb
JN030341	Singapore	2010	Cosmopolitan_Genotype_Ivb	JN030341_SINGAPORE_2010_Cos_Ivb
JN030342	Singapore	2010	Cosmopolitan_Genotype_Ivb	JN030342_SINGAPORE_2010_Cos_Ivb
JN196613	Singapore	2010	Cosmopolitan_Genotype_Ivb	JN196613_SINGAPORE_2010_Cos_Ivb
JN196614	Singapore	2010	Cosmopolitan_Genotype_Ivb	JN196614_SINGAPORE_2010_Cos_Ivb
JN544397	Singapore	2010	Cosmopolitan_Genotype_Ivb	JN544397_SINGAPORE_2010_Cos_Ivb
JN030343	Singapore	2010	Cosmopolitan_Genotype_Ivb	JN030343_SINGAPORE_2010_Cos_Ivb
JN030344	Singapore	2010	Cosmopolitan_Genotype_Ivb	JN030344_SINGAPORE_2010_Cos_Ivb
JN544398	Singapore	2011	Asian_I	JN544398_SINGAPORE_2011_Asian_I
JN544399	Singapore	2011	Cosmopolitan_Genotype_Iva	JN544399_SINGAPORE_2011_Cos_Iva
KT232041	Nepal	2013	Asian_II	KT232041_NEPAL_2013_Asian_II
KT232042	Nepal	2013	Cosmopolitan_Genotype_Iva	KT232042_NEPAL_2013_Cos_Iva
KT232043	Nepal	2013	Cosmopolitan_Genotype_Iva	KT232043_NEPAL_2013_Cos_Iva
KT232044	Nepal	2013	Cosmopolitan_Genotype_Iva	KT232044_NEPAL_2013_Cos_Iva
KT232045	Nepal	2013	Asian_II	KT232045_NEPAL_2013_Asian_II

KT232046	Nepal	2013	Cosmopolitan_Genotype_Iva	KT232046_NEPAL_2013_Cos_Iva
KT232047	Nepal	2013	Cosmopolitan_Genotype_Iva	KT232047_NEPAL_2013_Cos_Iva
KT232048	Nepal	2013	Asian_II	KT232048_NEPAL_2013_Asian_II
KT232049	Nepal	2013	Cosmopolitan_Genotype_Iva	KT232049_NEPAL_2013_Cos_Iva
KT232050	Nepal	2013	Cosmopolitan_Genotype_Iva	KT232050_NEPAL_2013_Cos_Iva
KT232051	Nepal	2013	Cosmopolitan_Genotype_Iva	KT232051_NEPAL_2013_Cos_Iva
KT232052	Nepal	2013	Cosmopolitan_Genotype_Iva	KT232052_NEPAL_2013_Cos_Iva
KT232053	Nepal	2013	Asian_II	KT232053_NEPAL_2013_Asian_II
KT232054	Nepal	2013	Asian_II	KT232054_NEPAL_2013_Asian_II
KT232055	Nepal	2013	Asian_II	KT232055_NEPAL_2013_Asian_II
L10041	Brazil	1990	Asian_I	L10041_BRAZIL_1990_Asian_I
L10042	Burkina Faso	1983	Cosmopolitan_Genotype_Iva	L10042_BURKINAFASO_1983_Cos_Iva
L10043	India	1957	Asian_American	L10043_INDIA_1957_Asian_American
L10045	Philippines	1983	Asian_II	L10045_PHILIPPINES_1983_Asian_II
L10051	Somalia	1984	Cosmopolitan_Genotype_Iva	L10051_SOMALIA_1984_Cos_Iva
L10054	Srilanka	1990	Cosmopolitan_Genotype_Ivb	L10054_SRILANKA_1990_Cos_Ivb
M15075	Jamaica	1986	Asian_I	M15075_JAMAICA_1986_Asian_I
M20558	Jamaica	1988	Asian_I	M20558_JAMAICA_1988_Asian_I
M24450	Srilanka	1989	Asian_II	M24450_SRILANKA_1989_Asian_II
M29095	New Guinea	1944	Asian_II	M29095_NEW GUINEA_1944_Asian_II
U87411	Thailand	1984	Asian_I	U87411_THAILAND_1984_Asian_I
MK209641	Nepal	2017		Nep48_D2
MK209642	Nepal	2017		Nep49_D2
MK209643	Nepal	2017		Nep50_D2
MK209644	Nepal	2017		Nep9_D2

Supplementary Table S4. District and province wise dengue infections, serological tests in the year 2017 and 2018.

Supplementary Table – 4. District and province wise dengue infections, serological tests in the year 2017 and 2018.

Districts	Province	Cases	Geograph Region	Above sea level (mt)	Infections No. (%)			NS1 +ve			IgM +ve			IgG +ve			Primary Infection			Secondary			Seronegative			Grand Total		
					2017	2018	Total	2017	2018	Total	2017	2018	Total	2017	2018	Total	2017	2018	Total	2017	2018	Total	2017	2018	Total	2017	2018	Total
Kapilvastu	Lumbini	1	Terai	<300-2000	1 (2.12%)	-	1 (2.12%)	1		1	1		1	1		1	1		1	0		0	0		0	1	0	1
Rupandehi	Lumbini	9	Terai	100-1229	9 (19.15%)	-	9 (19.15%)	7		7	7		7	3		3	7		7	1		1	1		1	9	0	9
Nawalpur#	Gandaki	1	Terai	<300-2000	1 (2.12%)	-	1 (2.12%)	1		1	1		1	0		0	1		1	0		0	0		0	1	0	1
Jhapa	Koshi	2	Terai	<300-1000	2 (4.26%)	-	2 (4.26%)	2		2	1		1	0		0	1		1	1		1	0		0	2	0	2
Bardiya	Lumbini	1	Terai	<300-2000	1 (2.12%)	-	1 (2.12%)	1		1	0		0	0		0	1		1	0		0	0		0	1	0	1
Sarlahi	Madhesh	10	Terai	<300-1000	10(21.27%)	-	10(21.27%	8		8	4		4	7		7	1		1	8		8	1		1	10	0	10
Kailali	Sudur Paschim	1	Terai	179-1957	1 (2.12%)	-	1 (2.12%)	1		1	0		0	0		0	0		0	0		0	1		1	1	0	1
Rautahat	Madhesh	2	Terai	<300-1000	1 (2.12%)	1(7.14%)	2 (3.28%)	1	1	2	0		0	1		1	0	1	1	1		1	0		0	1	1	2
Chitwan*	Bagmati	7	Terai	<300-2000	6 (12.77%)	1(7.14%)	7(11.46%)	6	1	7	4	1	5	1		1	4		4	0	1	1	2		2	6	1	7
Dhading*	Bagmati	6	Hill	488-7809	6 (12.77%)	-	6 (12.77%)	6		6	4		4	2		2	3		3	2		2	1		1	6	0	6
Makawanpur*	Bagmati	4	Hill	<300-3000	4 (8.51%)	-	4 (8.51%)	3		3	3		3	3		3	2		2	2		2	0		0	4	0	4
Nuwakot*	Bagmati	3	Hill	300-5000	2 (4.26%)	1(7.14%)	3 (4.9%)	0	1	1	2		2	1		1	2		2	0		0	0	1	1	2	1	3
Tanahun*	Gandaki	1	Hill	<300-2000	1 (2.12%)	-	1 (2.12%)	1		1	1		1	0		0	1		1	0		0	0		0	1	0	1
Kaski*	Gandaki	3	Hill	300-6400	2 (4.26%)	1(7.14%)	3 (4.9%)	2	1	3	1	1	2	1	1	2	1		1	1	1	2	0		0	2	1	3
Ramechhap	Bagmati	1	Hill	-	-	1(7.14%)	1(7.14%)		1	1			0			0			0			0		1	1	0	1	1
Syangja	Gandaki	2	Hill	-	-	2(14.29%)	2(14.29%)		2	2		1	1		1	1			0		1	1		1	1	0	2	2
Kathmandu	Bagmati	7	Hill	-	-	7(50%)	7(50%)		7	7		5	5		2	2		5	5			0		2	2	0	7	7
		61		Total	47	14	61	40	#	54	29	8	37	20	4	#	25	6	31	16	3	19	6	5	11	47	14	61

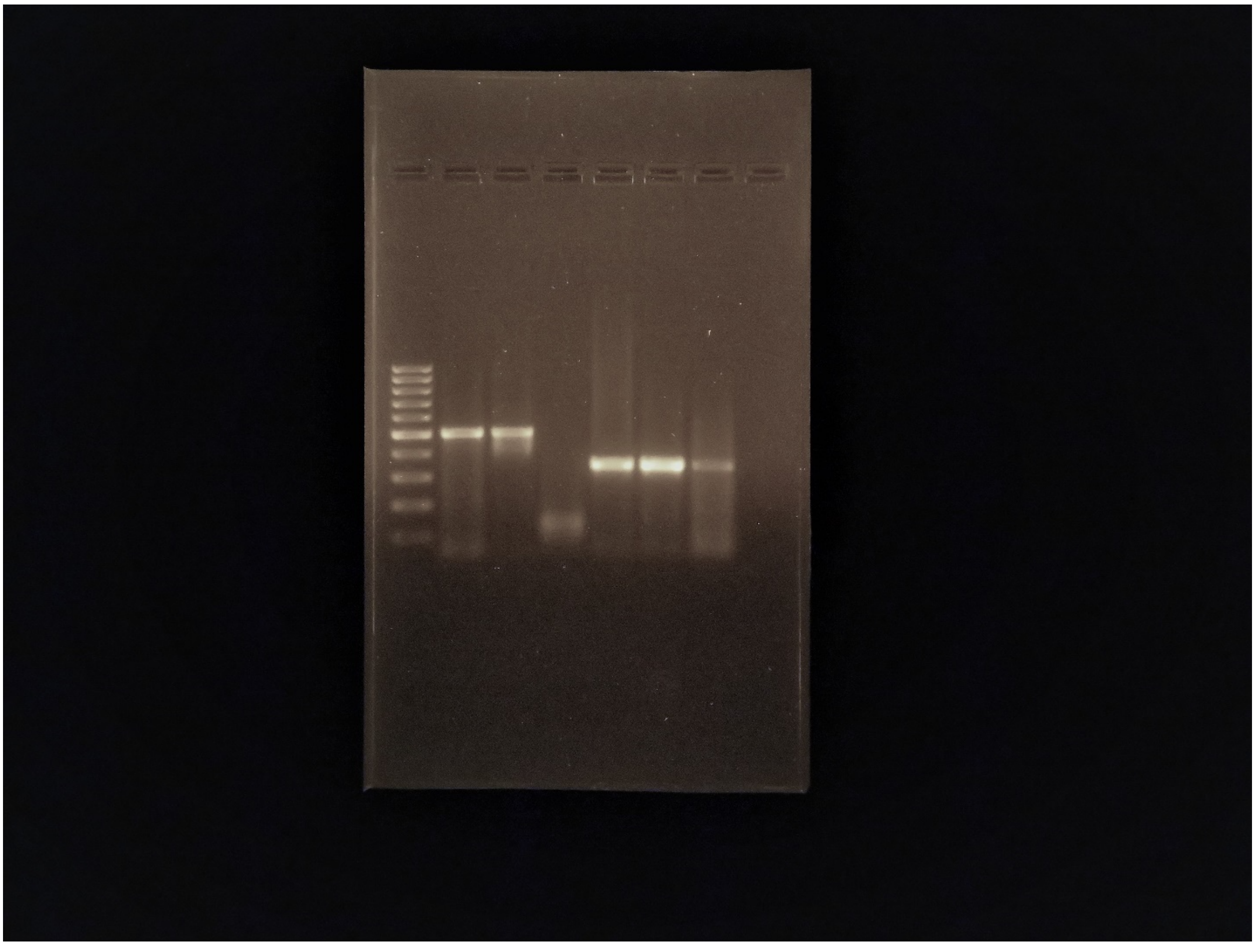

Supplementary Figure S1. Gel image under UV Transilluminator of the second round nested PCR of envelope gene that gave 500 bp, 337 bp and 189 bp amplicon corresponding to the expected size of DENV1, DENV2 and DENV3 respectively. 100bp ladder [Thermo Scientific Generuler 100 bp DNA ladder, ready to use (Cat. No. #SM0243)] was run along with the PCR product.

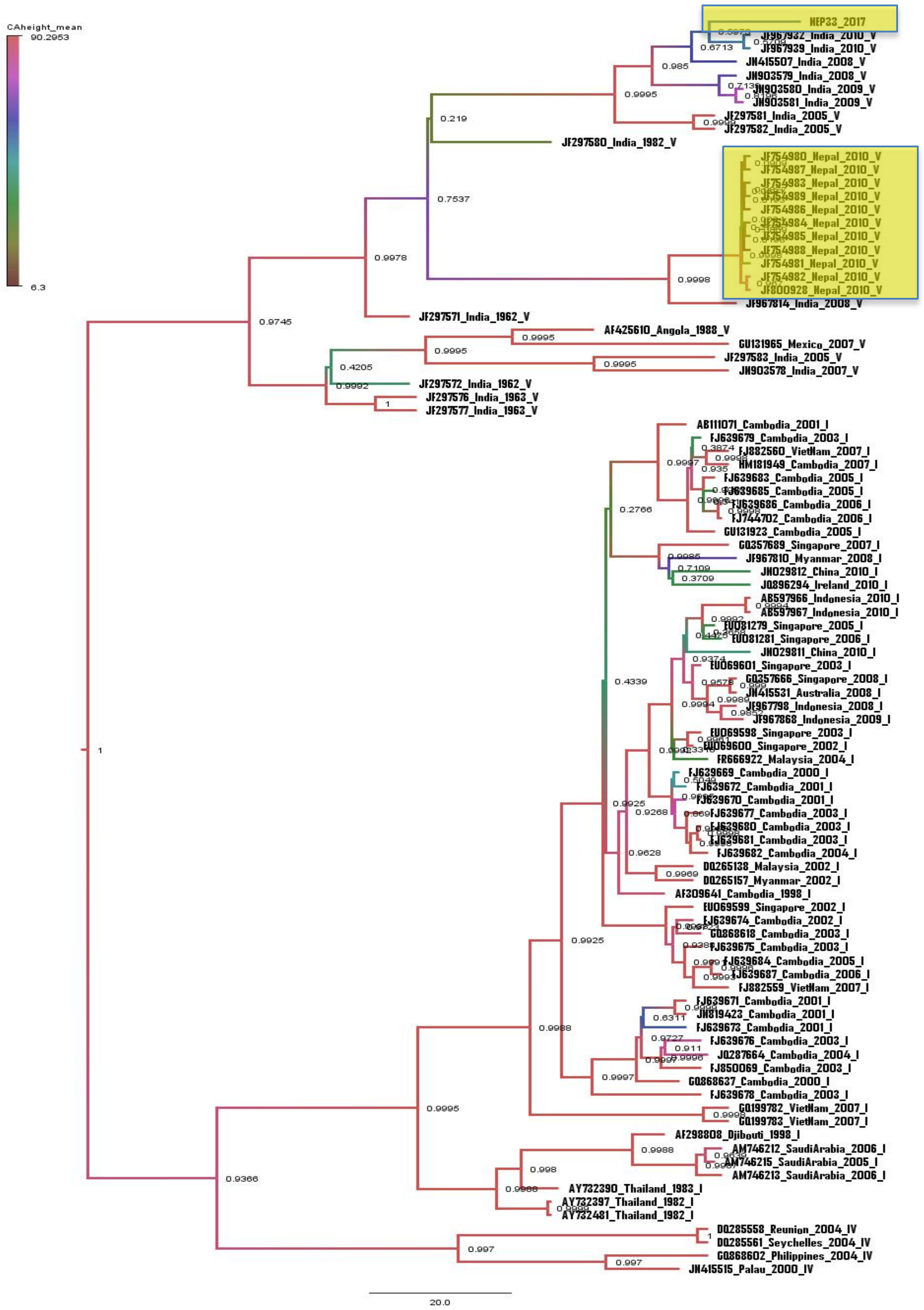

Supplementary Figure S2 Phylogeny tree of DENV1 serotype constructed using BEAST v2.5.1 using Gamma+I+T93 substitution model against Dengue genotyping database. All the sequences in the tree are labelled as; accession no._country_year of isolation_genotype. Tree are labelled with posterior probability at node and branch time indicates the length of the respective branch. Branch color is also highlighting posterior probability with a color gradient as indicated in the legends.

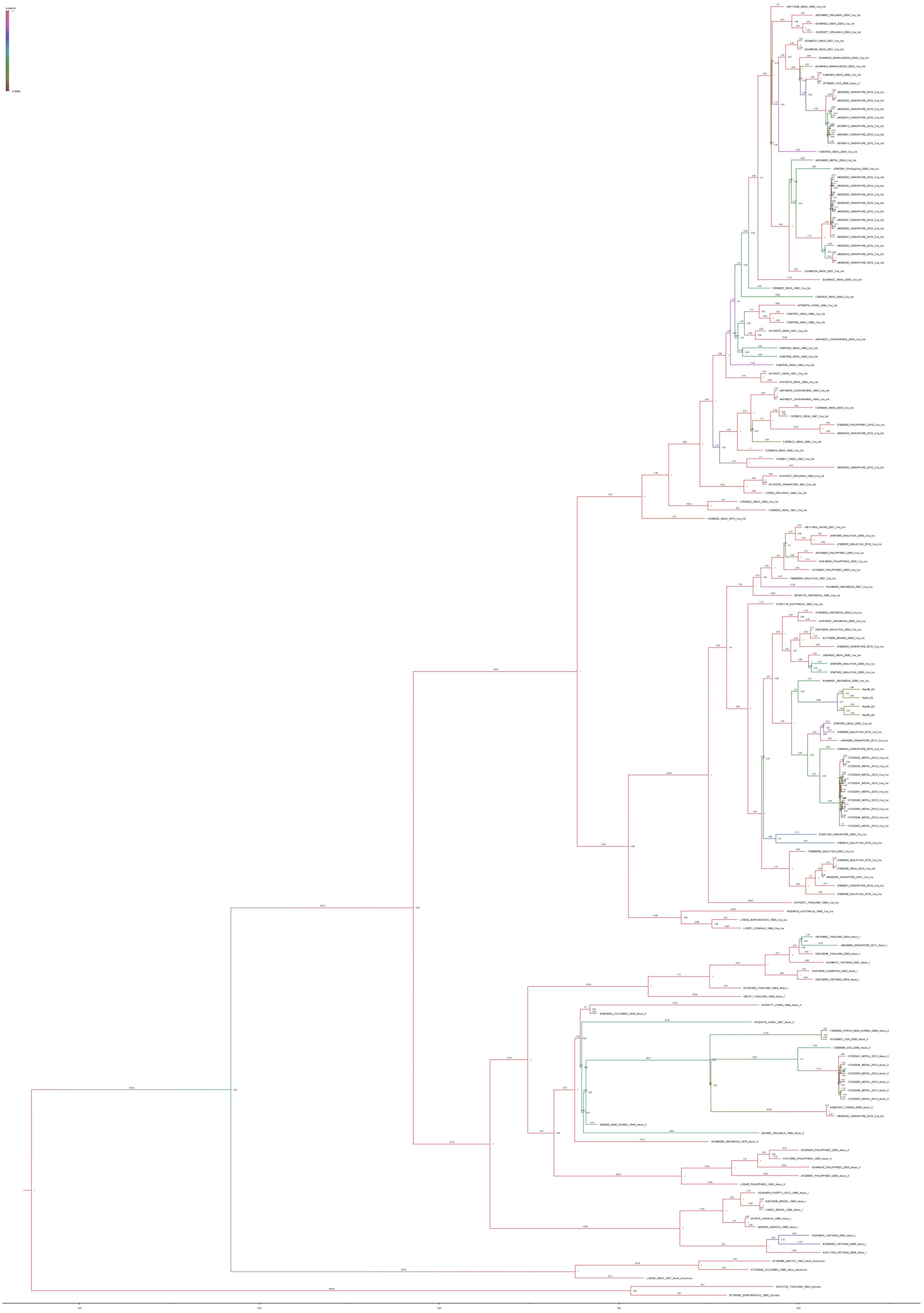

Supplementary Figure S3 Phylogeny tree of DENV2 genotype constructed using BEAST v2.5.1 using Gamma+I+T93 substitution model against Dengue genotyping database. All the sequences in the tree are labelled as; accession no._country year of isolation genotype. Tree are labelled with posterior probability at node and branch time indicates the length of the respective branch. Branch color is also highlighting posterior probability with a color gradient as indicated in the legends.

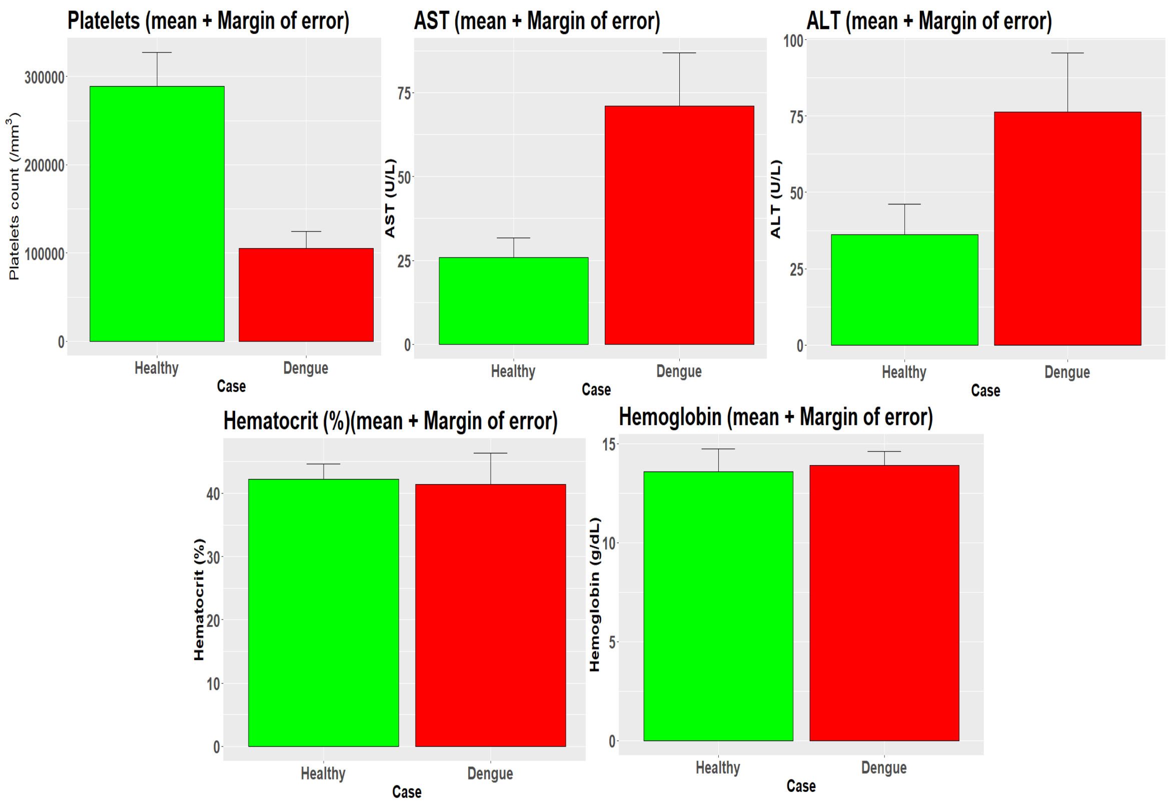

Supplementary Figure S4: Summary statistics of platelets, AST, ALT, hematocrit and hemoglobin for healthy versus Dengue patients.

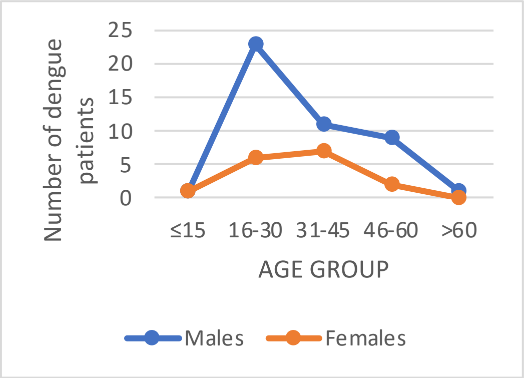

Supplementary Figure S5: Sex and age wise distribution of dengue cases in the year 2017 and 2018 (n=61) (The age of patients ranged from 12 to 74 years).
